# Improved chromosome-level genome assembly of *Pleurotus ostreatus* CCMSSC 00389

**DOI:** 10.1093/g3journal/jkag150

**Published:** 2026-06-11

**Authors:** Yanjiao Ma, Chenyang Huang, Mengran Zhao, Zeyin Wu, Fangjie Yao, Lijiao Zhang

**Affiliations:** College of Horticulture, Jilin Agricultural University, 2888 Xincheng Street, Changchun, Jilin 130118, China; State Key Laboratory of Efficient Utilization of Arable Land in China, Institute of Agricultural Resources and Regional Planning, Chinese Academy of Agricultural Sciences, 12 Zhongguancun South Street, Beijing 100081, China; State Key Laboratory of Efficient Utilization of Arable Land in China, Institute of Agricultural Resources and Regional Planning, Chinese Academy of Agricultural Sciences, 12 Zhongguancun South Street, Beijing 100081, China; State Key Laboratory of Efficient Utilization of Arable Land in China, Institute of Agricultural Resources and Regional Planning, Chinese Academy of Agricultural Sciences, 12 Zhongguancun South Street, Beijing 100081, China; College of Horticulture, Jilin Agricultural University, 2888 Xincheng Street, Changchun, Jilin 130118, China; State Key Laboratory of Efficient Utilization of Arable Land in China, Institute of Agricultural Resources and Regional Planning, Chinese Academy of Agricultural Sciences, 12 Zhongguancun South Street, Beijing 100081, China; College of Horticulture, Jilin Agricultural University, 2888 Xincheng Street, Changchun, Jilin 130118, China; State Key Laboratory of Efficient Utilization of Arable Land in China, Institute of Agricultural Resources and Regional Planning, Chinese Academy of Agricultural Sciences, 12 Zhongguancun South Street, Beijing 100081, China

**Keywords:** *Pleurotus ostreatus*, whole-genome sequencing, chromosome-level genome, PacBio Sequel II sequencing, functional annotation, comparative genomic analysis

## Abstract

*Pleurotus ostreatus* is one of the major cultivated edible mushrooms in China, and possesses high nutritional value and prominent medicinal properties. A chromosome-level genome assembly of this species is therefore of great significance for both basic biological research and industrial applications. In this study, we present a chromosome-level genome assembly of the commercial *P. ostreatus* cultivar CCMSSC 00389, which shows improved continuity and completeness relative to previously published genome assemblies. The new assembly genome consists of 12 pseudo-chromosomes with a total length of 40.39 Mb. Compared with previous versions, the total genome size increased by 11.31%, the number of scaffolds was reduced from 2,529 to 20, and the scaffold N50 length was improved 9.53 fold. Accordingly, the average gene length increased from 1,523 to 1,867.5 bp, and the number of genes functionally annotated in public databases rose from 11,095 to 12,514. BUSCO analysis was performed to evaluate the integrity and completeness of the newly assembled genome. A total of 269 complete BUSCO genes were identified, accounting for 92.76% of the total assessed genes, demonstrating the acceptable completeness of the new assembled genome. This high-quality genome provides a crucial foundation for exploring evolutionary dynamics, physiological and trait regulatory mechanisms, as well as molecular breeding studies in *P. ostreatus*.

## Introduction

*Pleurotus ostreatus* is a highly nutritious edible and medicinal mushroom (Ling et al. 2025). With the rapid expansion of the edible mushroom industry, increasing emphasis has been placed on genetic breeding, underscoring the importance of elucidating its underlying genetics. Chromosome-level genome assemblies are a critical tool for studying the genetics of edible mushrooms, enabling in-depth elucidation of their biology and key agronomic traits.

Researchers identified a key variant block on chromosome 9 of *Lentinula edodes* associated with thermo-tolerant trait for fruiting body formation, and this finding facilitated the development of molecular markers for breeding (Yoo et al. 2024). In *Flammulina filiformis*, the whole genome was sequenced and assembled. Multi-omics analysis was employed to screen out the pathways and genes related to the low-temperature response, and further analyze the expression profile of the carbohydrate-active enzyme (CAZyme) gene, which are crucial for the low-temperature adaptation of *F. filiformis* (Liang et al. 2025). The whole genome of *Laetiporus sulphureus* NWAFU-1, a wild strain isolated from the Qinling Mountains in China, was sequenced and assembled. And candidate genes associated with mating type, polysaccharide synthesis, carbohydrate-active enzymes, and secondary-metabolite biosynthesis were identified (Dong et al. 2022).

The emergence of the third-generation sequencing technology has driven substantial progress in fungal genomic research. Compared with conventional sequencing platforms, it features longer read lengths, better assembly continuity and higher accuracy, enabling the acquisition of high-quality, chromosome-level fungal genome assemblies (van Dijk et al. 2018). Driven by continuous technological advancements and reduced sequencing costs, genomic studies on *P. ostreatus* have expanded rapidly, with 9 genome assemblies currently available in the publicly database (Table 1).

**Table 1.** Summary of genomic assembly information of *P. ostreatus.*

Species name	Strain	Assembly type	Genome size (Mb)	Predicted gene number	Scaffold number	Scaffold N50 (Mb)	Sequencing technology	Source	Accession number
*P. ostreatus*	PC9	haploid	34.9	11,847	16	3.5	PacBio; Illumina	NCBI	JACETU000000000 (Lee et al. 2021)
*P. ostreatus*	PC9.15	haploid	35.3	13,556	13	3.3	Nanopore	NCBI	JAQZRA000000000.1 (Lee et al. 2026)
*P. ostreatus*	PC80	haploid	40.5	14,374	14	3.6	Illumina, PacBio	NCBI	JBMSAL000000000 (Wu et al. 2025)
*P. ostreatus*	CCMSSC 03989	haploid	34.4	10,696	89	2.9	Illumina	NCBI	QLNW00000000 (Wang et al. 2018)
*P. ostreatus*	CCMSSC 00389	haploid	35.1	10,955	136	3.1	Illumina	NCBI	MAYC00000000.2 (Wang et al. 2019)
*P. ostreatus*	CCMSSC 00389	haploid	35.8	13,438	2,529	0.39	Illumina HiSeq 2500	NCBI	MAYC00000000 (MAYC00000000.1) (Qu et al. 2016)
*P. ostreatus*	PC15	haploid	34.3	12,460	12	3.3	Sanger dideoxy sequencing	NCBI	AYUK00000000 (Riley et al. 2014)
*P. ostreatus*	595	haploid	79.7	…	329	0.36	Illumina HiSeq, Oxford Nanopore	NCBI	JAKNCV000000000 (Shao et al. 2022)
*P. ostreatus*	DSM 11191	dikaryotic	66.0	12,914	214	0.62	PacBio	JGI	PleosDSM11191_1

The *P. ostreatus* strain CCMSSC 00389 from China Center for Mushroom Spawn Standards and Control (CCMSSC) is a widely cultivated strain and is frequently used as a test strain in genetic breeding research. However, no chromosome-level genome of this strain is available. Therefore, this study employed PacBio Sequel II long-read sequencing and Hi-C chromatin conformation capture technology to successfully assemble its genome, thus providing a reliable reference sequence for future research. Compared with previous versions, this newly assembled chromosome-level genome exhibits improved continuity and integrity, providing a foundation for elucidating the molecular mechanisms underlying major agronomic traits and advancing marker-assisted breeding.

## Materials and methods

### Strain and experimental materials

The *P. ostreatus* strain CCMSSC 00389 (hereinafter 00389) is preserved in the China Center for Mushroom Spawn Standards and Control. The strain was cultured on potato dextrose agar medium at 28 °C in the dark. After 7 d of cultivation, mycelia were collected for genomic DNA extraction.

### PacBio and Hi-C library construction, quality inspection, and sequencing

The integrity, purity, and concentration of the extracted DNA were evaluated using agarose gel electrophoresis, Nanodrop spectrophotometer, and Qubit fluorometer, respectively. Qualified DNA samples were then used for library construction and the resulting high-quality library was sequenced on PacBio Sequel II platform in circular consensus sequencing (CCS) sequencing mode.

Hi-C libraries were constructed in parallel to capture chromatin interaction data for chromosome-level genome assembly. Then the library concentration and insert size were detected using Qubit and Agilent 2100, respectively. To ensure library quality, concentration of constructed libraries was accurately determined using qPCR. Following quality validation, paired-end high-throughput sequencing was performed using the Illumina platform with a sequencing read length of PE150 and a target data volume ≥6 Gb.

### Genome assembly and BUSCO analysis

To generate high-accuracy CCS reads, PacBio raw data was processed using the CCS program in SMRT Link (v11.0) (parameters: –min-passes 5 –min-rq 0.9). The CCS reads were assembled using Hifi-asm (v0.16.1) (Cheng et al. 2021). Then, the assembled genome was corrected using Pilon (v1.24) (Walker et al. 2014) to obtain a more accurate genome assembly. To obtain high-quality data, Hi-C sequencing data were then evaluated and filtered using HiC-Pro (v3.1.0) (Servant et al. 2015). BWA was used for reads alignment (Li et al. 2010). Finally, the genome sequence was clustered, ordered, and oriented into chromosome groups using LACHESIS (Burton et al. 2013). The completeness of the genome assembly results was assessed using BUSCO (v2.0) (Simão et al. 2015) against the fungal database fungi OrthoDB version 9 (fung_odb9).

### Repeat sequence prediction

We used LTR_FINDER (v1.0.5) (Xu and Wang 2007), MITE-Hunter (v2010) (Han and Wessler 2010), RepeatScout (v1.0.5) (Price et al. 2005), and PILER-DF (v2.4) (Edgar and Myers 2005) to construct a repeat sequence database of the *P. ostreatus* genome based on structure and *de novo* prediction. Then this repeat sequence database was classified using Pseudo Agent System for Transposable Elements Classification (PASTEClassifier) (Wicker et al. 2007). Subsequently, the classified database was merged with the Repeat Base Database (Repbase‌) (Jurka et al. 2005) to construct the final repeat sequence database. Finally, the repeat sequence in the *P. ostreatus* 00389 genome were predicted using RepeatMasker (v4.0.6) (Tarailo-Graovac and Chen 2009) based on this final database.

### Coding genes, noncoding RNAs, and pseudogene prediction

Gene structure prediction includes *de novo* and homologous protein prediction. The *de novo* prediction was performed using Genscan (Burge and Karlin 1997), Augustus (v2.4) (Stanke and Waack 2003), GlimmerHMM (v3.0.4) (Majoros et al. 2004), GeneID (v1.4) (Alioto et al. 2018), and SNAP (v2006) (Korf 2004). The homologous protein prediction was performed using GeMoMa (v1.3.1) (Keilwagen et al. 2019). Both prediction results were integrated using EvidenceModeler (v1.1.1) (Haas et al. 2008). tRNAs were predicted using tRNAscan-SE (v1.3.1) (Lowe and Eddy 1997). rRNAs and other noncoding RNAs were predicted using Infernal (v1.1.1) (Nawrocki and Eddy 2013).

Pseudogenes were identified following the procedures below. First, predicted protein sequences were aligned against the SwissProt database using GenBlastA v1.0.4 to screen homologous gene sequences (She et al. 2009). Then GeneWise v2.4.1 was then used to detect premature stop codons and frameshift mutations within these homologous sequences for pseudogene identification (Birney et al. 2004).

### Genome function annotation

Predicted proteins were annotated by BLAST alignment against eukaryotic Orthologous Groups (KOG) (Tatusov et al. 2000), Kyoto Encyclopedia of Genes and Genomes (KEGG) (Kanehisa et al. 2004), Swiss-Prot Sequence Database‌ (SwissProt) (Boeckmann et al. 2003), Translation of EMBL (TrEMBL) (Boeckmann et al. 2003), and Nonredundant Database (NR) (Yang et al. 2025). Based on NR database comparisons, Blast2GO (Conesa et al. 2005) performs functional annotation of the Gene Ontology (GO) (Ashburner et al. 2000) database. The software HMMER (v3.3.2) (Eddy 1998) was used to annotate Protein Families (Pfam) (Finn et al. 2016) functions.

Based on the predicted gene protein sequences, we performed a BLAST alignment with functional databases such as the Transporter Classification Database (TCDB) (Saier et al. 2006), Pathogen-Host Interactions database (‌PHI) (Winnenburg et al. 2006), Cytochrome P450 Engineering Database (CYPED) (Fischer et al. 2007), and Database of Known Fungal Virulence Factors ‌(DFVF) (Lu et al. 2012). In addition, functional annotation of CAZyme genes was performed using HMMER (v3.3.2) (Eddy 1998) software based on Carbohydrate-Active EnZYmes Database (CAZy) (Cantarel et al. 2009).

### Gene cluster prediction and protein subcellular localization analysis

Biosynthetic gene clusters were identified using anti-SMASH (v6.0.0) (Blin et al. 2021). To find the proteins containing the signal peptide, all predicted protein sequences were analyzed using SignalP (v4.0) (Petersen et al. 2011). To identify proteins containing transmembrane helices, protein sequences were analyzed using TMHMM (v2.0) (Krogh et al. 2001). Proteins containing a transmembrane helix were excluded from those with predicted signal peptides; the remaining proteins were classified as secreted proteins and subsequently analyzed using EffectorP (v3.0) (Sperschneider et al. 2016) for fungal effector prediction.

### Genome analysis and comparison

The newly assembled chromosome-level genome of *P. ostreatus* 00389 was compared with its previously published genome assembly. For synteny analysis, we selected scaffolds >10 kb from the previous genome assembly and compared them with the newly assembled genome. Synteny analysis was performed using Nucmer (v4.0.1), and alignments with ≥95% sequence identity were retained. The results were visualized with Circos (v0.69-8). In addition, 7 aspects were compared, namely: genome sequencing data, genome assembly results, repeat sequence prediction results, coding gene prediction results, noncoding RNA and pseudogene prediction results, functional gene annotation results, and gene cluster prediction and protein subcellular localization.

## Results and discussion

### Improved chromosomal genome assembly of strain 00389 using PacBio Sequel II and Hi-C

The chromosome-level genome of strain 00389 was assembled using PacBio Sequel II sequencing and Hi-C technologies. Compared with the previous version, the final chromosome-level genome of strain 00389 exhibited substantial improvements in both quality and length distribution. The number of sequencing reads decreased from 25,877,469 to 282,123 (98.91% decrease), with the total number of bases declining from 5.23 to 2.33 Gb (55.45% decrease). The average read length increased from 101 to 8,256 bp, and the maximum read length extended from 101 to 36,782 bp. The average and maximum read lengths increased 81.74-fold and 364.18-fold, respectively. The read N50 of the final sequencing data was 10,211 bp (Table 2). Longer read lengths were obtained with PacBio sequencing, which can span multiple repeat units and capture large fragments (Logsdon et al. 2020). In addition, the mapping efficiency of Hi-C reads was 95.27%, with 92% of reads showing unique mapping (Table 3). These high mapping efficiency and unique alignment rate further validate the completeness and accuracy of the assembled genome.

**Table 2.** Sequencing data statistics of the 2 genome assemblies.

Data type	00389_original	00389_final
SeqNum	25,877,469	282,123
SumBase (G)	5.23	2.33
N50Len (bp)	…	10,211
MeanLen (bp)	101	8,256
MaxLen (bp)	101	36,782

**Table 3.** Hi-C read mapping efficiency and uniqueness.

Mapping type	00389_final	
	Number	Ratio (%)
Total read pairs	22,822,880	100
Mapped reads	43,488,307	95.27
Unique mapped read pairs	20,996,853	92

### Completeness evaluation and comparative analysis of genome assembly quality

These high-quality reads were assembled into a 40.39 Mb genome, representing a 12.76% increase compared with the previous 35.82 Mb version. The scaffolds N50 (>1 kb) exhibited a 9.53-fold increase, from 0.39 to 3.76 Mb, concomitant with a reduction in scaffold number from 2,529 to 20. The contig N50 showed a 22.54-fold improvement, rising from 0.10 to 2.24 Mb, while the number of contigs decreased from 3,505 to 38. (Table 4).

**Table 4.** Comparative analysis of genome assembly quality.

Data type	00389_original	00389_final
Scaffold length (bp)	35,820,573	40,393,584
Scaffold number	2,529	20
Scaffold N50 (bp)	394,787	3,763,286
Scaffold N90 (bp)	61,660	2,101,780
Contig length (bp)	34,872,813	40,391,784
Contig number	3,505	38
Contig N50 (bp)	99,605	2,244,706
Contig N90 (bp)	12,993	819,672
GC content (%)	49.54	51.04
Gaps number	…	18

Using Hi-C–assisted genome assembly technology, 40.24 Mb (99.62%) of the genome was anchored into 12 pseudo-chromosomes, 6 of which possess complete telomere-to-telomere structures (Supplementary Table 1). However the Hi-C interaction heatmap clearly distinguished 12 groups, showing strong intra-chromosomal interactions and negligible inter-chromosomal signals (Supplementary Fig. 1). This result confirmed the high accuracy of the Hi-C-assisted assembly and demonstrated that all scaffolds were correctly clustered, ordered, and oriented into 12 pseudo-chromosomes.

**Fig. 1.**
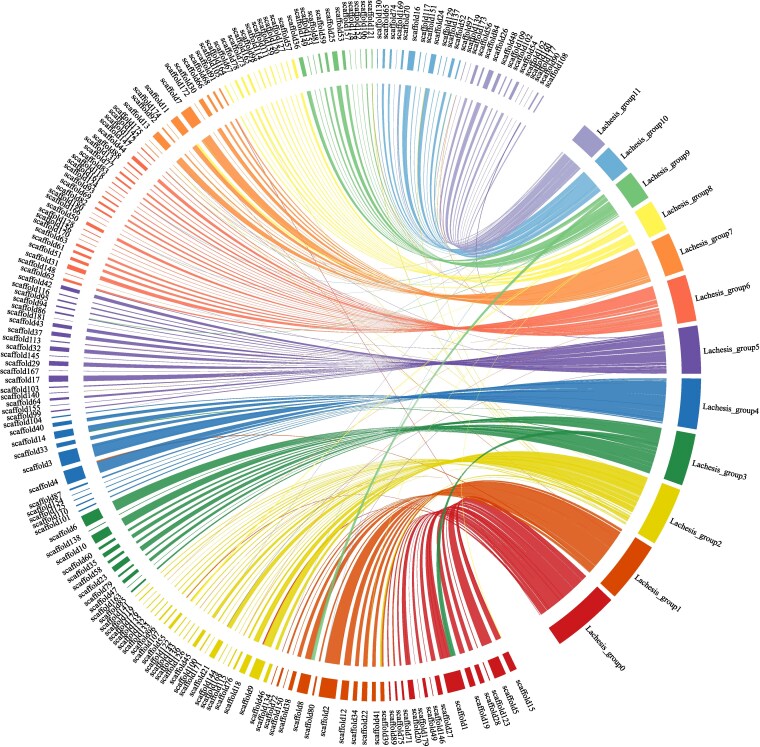
Genome collinearity between the 2 genomic assemblies of the 00389 strain. The left arc displays scaffolds derived from the earlier genome assembly, whereas the right arc corresponds to chromosomes of the newly assembled genome.

Moreover, synteny analysis was performed between the 2 genome assemblies. The results showed that the continuity and quality of the new assembled genome was improved (Fig. 1). A synteny analysis showed all scaffolds >10 kb from the previous assembly were successfully anchored into 12 Lachesis groups (corresponding to 12 pseudo-chromosomes) with correct order and orientation. Each Lachesis_group contained multiple scaffolds from previous assembled genome. For instance, 14 scaffolds from previous genome were continuously mapped to Lachesis_group0 of the new genome and cover its full length (4.99 Mb). Those results demonstrate a high degree of collinearity between the new genome and the previous scaffold-level genome.

The integrity of the newly assembled genome was evaluated using BUSCO analysis. The results showed that the new assembled genome contained 269 complete BUSCO genes out of 290 (92.76%), including 264 single-copy genes (91.03%), 5 duplicated genes (1.72%), 7 fragmented genes (2.41%), and 14 missing genes (4.83%) (Table 5). It is slightly below the highest-quality assemblies typically obtained with HiFi-based approaches (>95%). These results reflect that the chromosome-level genome achieves acceptable standards of completeness and quality, providing a valuable genomic resource for subsequent genetic research and molecular breeding studies in *P. ostreatus*.

**Table 5.** BUSCO evaluation of the final genome assembly.

Type	00389_final	
	Number	Percentage (%)
Complete BUSCOs (C)	269	92.76%
Complete and single-copy BUSCOs (S)	264	91.03%
Complete and duplicated BUSCOs (D)	5	1.72%
Fragmented BUSCOs (F)	7	2.41%
Missing BUSCOs (M)	14	4.83%
Total Lineage BUSCOs	290	100%

### Variations in repetitive sequence composition between the newly assemble genome and previous versions

Comparative analysis of repetitive sequences was conducted between the previous and newly assembled genomes. The number of predicted repeat sequences increased from 106 to 3,703, and their total length expanded from 13,583 to 5,662,971 bp, raising their genomic proportion from 0.04% to 14.02%. These results were more consistent with the typical repeat content of fungal genomes (Dutta 1974). Repeat sequences annotation results revealed significant differences in the repeat landscape between the 2 genome assemblies. In the previous genome, the predominant repeat types were terminal inverted repeats, unknown sequences, and simple sequence repeats. Whereas in the new genome, Class I retrotransposons represented the predominant repetitive component, exceeding Class II DNA transposons by more than 6-fold. Among Class I retrotransposons, the long terminal repeat retrotransposons LTR/Gypsy were the most abundant, comprising 1,552 copies and accounting for 7.57% of the new genome (Table 6). In addition, 1.56% of the sequences were classified as unknown repeats. The presence of unknown repetitive sequences likely reflects the limited coverage of current repeat databases. Overall, the new chromosome-level genome significantly improved the assembly integrity of repetitive regions and enabled more comprehensive and accurate annotation of transposable elements.

**Table 6.** Classification and comparison of repetitive sequence in the 2 genome versions.

	00389_original			00389_final		
Data type	Number	Length (bp)	Percentage (%)	Number	Length (bp)	Percentage (%)
Class I	0	0	0.00	2,930	4,459,687	11.04
DIRS	…	…	…	318	354,537	0.88
LINE	0	0	0.00	65	26,154	0.06
LTR	0	0	0.00	1	64	0.00
LTR/Copia	0	0	0.00	290	376,563	0.93
LTR/Gypsy	0	0	0.00	1,552	3,058,833	7.57
PLE|LARD	…	…	…	699	701,217	1.74
TRIM	…	…	…	5	2,932	0.01
Class II	9	3,790	0.01	466	437,768	1.08
Helitron	0	0	0.00	192	211,759	0.52
MITE	…	…	…	20	16,013	0.04
TIR	8	3,759	0.01	246	209,674	0.52
Unknown	1	31	0.00	8	498	0.00
PotentialHostGene	…	…	…	288	213,927	0.53
SSR	97	9,793	0.03	19	4,475	0.01
Unknown	…	…	…	829	628,495	1.56
Total	106	13,583	0.04	3,703	5,662,971	14.02

DIRS, direct repeat sequence; LINE, long interspersed repeated sequences; LTR, long terminal repeat sequence; LTR/Copia, copia transposon in long terminal repeat-retrotransposons; LTR/Gypsy, gypsy transposon in long terminal repeat-retrotransposons; PLE, plant-like element; LARD, large reverse transcription derived transposon; TRIM, tripartite motif; Helitron, transposon type; MITE, miniature inverted repeat transposable element; TIR, terminal inverted repeat; Unknown, unknown sequence; PotentialHostGene, potential host gene; SSR, simple sequence repeats.

### Changes in exons and introns of coding genes in the chromosome-level genome

Compared with the previous version, the number of predicted protein-coding genes decreased by 3.53%, from 13,438 to 12,964. By contrast, the total length of coding genes increased from 22.55 to 24.21 Mb and the average gene length increased from 1.68 to 1.87 kb. The total exon length increased from 17.73 to 18.34 Mb. In the previous genome, most genes were predicted with less exons, with an average of only 5.77 exons per gene and an average exon length of 228.40 bp. In the chromosome level genome, the average exon number per gene increased to 6.42, and the average exon length decreased to 220.23 bp. Overall, the average exon number of each gene were increased and the average exon length was decreased. This is consistent with exons being usually short and numerous in fungal genomes (Li et al. 2017). Furthermore, the total intron length increased from 4.86 to 5.87 Mp, accounting for 13.57 and 14.54% of the respective genomes. The average intron length increased from 73.59 to 83.57 bp. The number of introns increased from 66,045 to 70,295 and the average number of introns per gene slightly increased from 4.91 to 5.42 (Table 7). These results demonstrate that the newly assembled genome contains longer and more abundant intronic sequences, indicating the recovery of previously missing and truncated intronic regions in the earlier assembly. Collectively, the chromosome-level assembly of 00389 effectively removed redundant fragmented genes and optimized gene structural information, laying a more accurate foundation for subsequent gene function analysis and comparative genomic research.

**Table 7.** Statistical analysis of gene structural features.

Type	Item	00389_original	00389_final
Gene	Gene number	13,438	12,964
	Gene length (bp)	22,554,873	24,210,333
	Average gene length (bp)	1,678.44	1,867.50
Exon	Exon number	77,474	83,259
	Exon length (bp)	17,694,889	18,336,063
	Average exon length (bp)	228.40	220.23
	Average exon number	5.77	6.42
Intron	Intron number	66,045	70,295
	Intron length (bp)	4,859,984	5,874,270
	Average intron length (bp)	73.59	83.57
	Average intron number	4.91	5.42

### Noncoding RNA and pseudogene prediction

Compared with the previous version, the numbers of predicted noncoding RNAs (ncRNAs) and pseudogenes increased substantially (Table 8). The number of annotated ncRNAs increased from 196 to 290, reflecting substantially improved annotation of non-coding elements. The number of rRNAs increased from 2 to 44, while the number of rRNA families also expanded from 2 to 4. The number of tRNAs increased from 194 to 211. Additionally, 35 ncRNAs belonging to 12 families were newly predicted in the chromosome-level genome. These families fall into 4 functional categories: riboswitches involved in transcriptional regulation, spliceosomal components involved in pre-mRNA processing, RNAs associated with the translation machinery, and catalytic ribozymes. Overall, the new genome assembly exhibits a higher abundance and greater diversity of predicted ncRNAs, suggesting that *P. ostreatus* may possesses a comprehensive and diverse non-coding RNA-mediated regulatory system. Furthermore, the new assembled genome contained 238 pseudogenes with a total length of 546,245 bp, in contrast to merely 11 pseudogenes with total 874 bp in the previous version. Overall, the chromosome-level genome optimized the annotation of ncRNAs and pseudogenes.

**Table 8.** Statistics of noncoding RNA and pseudogenes.

Data type	00389_original	00389_final
Noncoding RNA number	196	290
Noncoding RNA family number	47	61
rRNA number	2	44
rRNA family number	2	4
tRNA number	194	211
tRNA family number	45	45
Other ncRNA number	…	35
Other ncRNA family number	…	12
Pseudogene number	11	238
Pseudogene size (bp)	874	546,245
Average pseudogene length (bp)	79.45	2,295.14

### Enhanced functional annotation of genes in the chromosome-level genome

**Fig. 2.**
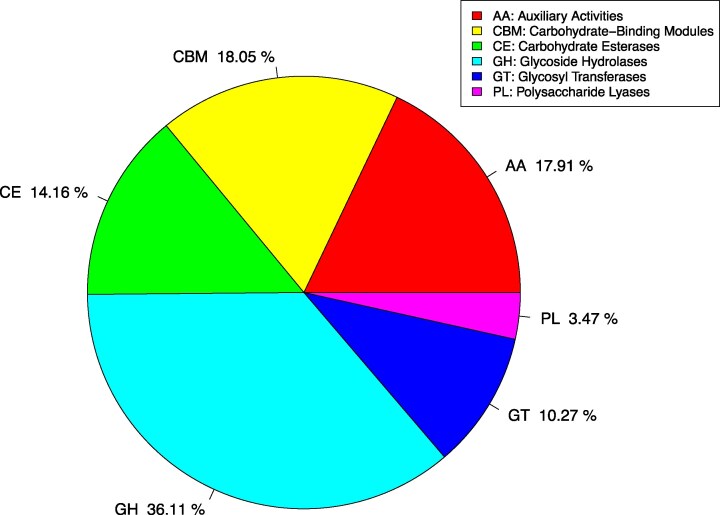
Composition of carbohydrate-active enzyme (CAZy) families in the chromosome-level genome assembly. Values represented the proportional contribution of each class of CAZyme to the total CAZyme repertoire.

**Fig. 3.**
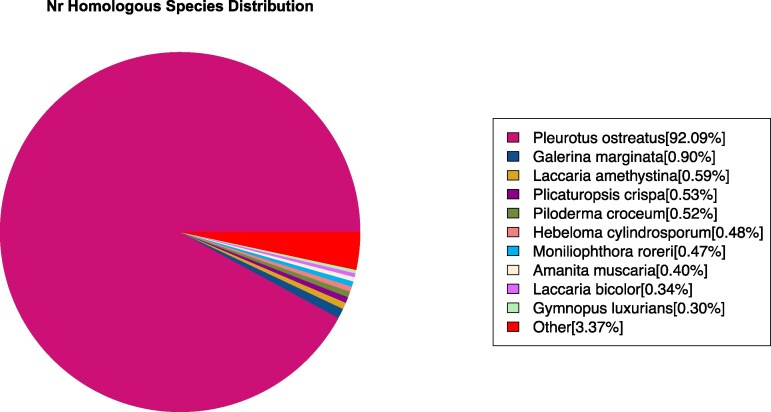
Taxonomic distribution of the BLAST results against the NR database. Different colors represent different homologous species.

In the previous assembled genome, all 13,438 predicted protein-coding genes were annotated against the KOG, COG, NOG, KEGG, GO, STRING, and NR functional databases using BLAST, achieving functional annotation of 11,095 genes (82.56%). In the new assembled genome, we performed the gene functional annotation using additional databases, including Pfam, TrEMBL, CAZyme, TCDB, PHI, CYPED, and DFVF. Based on all the databases mentioned above, 12,514 of the 12,964 predicted coding sequences (96.53%) were annotated. Gene functional annotation showed a comprehensive improvement in the new genome assembly, which may be attributed to the improved quality of the assembly and the expansion and continual updates of general protein sequence databases. Among the additional database, 2,815 genes (21.71%) were annotated to the PHI database, 2,003 (15.45%) to DFVF, 475 (3.66%) to CYPED, and 87 (0.67%) to TCDB. In addition, 627 genes (4.84%) were annotated and classified into CAZyme families based on CAZy database (Table 9). Further classification showed that glycoside hydrolases (GHs) accounted for the largest family with 260 genes (36.11%). Followed by carbohydrate-binding modules with 130 genes (18.05%), and auxiliary activities (AAs) with 129 genes (17.91%) (Fig. 2). These annotated results greatly enriched the dimensionality of gene functional characterization. Notably, annotation rates differed substantially among databases (Geistlinger et al. 2021). For instance, more than 96% of all predicted genes were successfully annotated to the TrEMBL and NR databases based on sequence alignment. Further analysis of NR annotation data revealed that 92.09% of the protein sequences exhibited homology to *P. ostreatus* (Fig. 3). In contrast, databases like GO, KEGG, and KOG, which rely on homology-based classification (Carbon et al. 2021), exhibit comparatively lower annotation rates, ranging from 25.96% to 56.19%.

**Table 9.** Comparative statistics of gene function annotation.

Type	Data_Type	00389_original	00389_final
General database	GO	6,566 (59.18%)	7,284 (56.19%)
	KEGG	3,254 (24.21%)	3,365 (25.96%)
	KOG	3,505 (26.08%)	5,470 (42.19%)
	Pfam	…	7,678 (59.23%)
	String	3,310 (24.63%)	…
	Swissprot	…	6,122 (47.22%)
	TrEMBL	…	12,505 (96.46%)
	NR	11,095 (82.56%)	12,496 (96.39%)
	All	11,095 (82.56%)	12,514 (96.53%)
Proprietary database	CAZyme	…	627 (4.84%)
	TCDB	…	87 (0.67%)
	PHI	…	2,815 (21.71%)
	CYPED	…	475 (3.66%)
	DFVF	…	2,003 (15.45%)

**Fig. 4.**
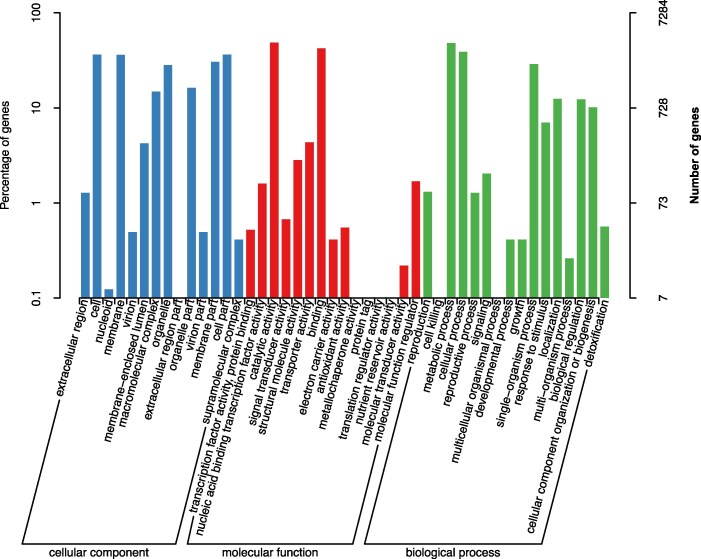
Classification statistics of GO functional annotation. The *x*-axis represents the 3 main GO categories. The left *y*-axis indicated the percentage of annotated genes and the right *y*-axis showed the number of genes in each category.

GO enrichment analysis categorized the annotated genes into 3 major categories: molecular function, cellular component, and biological process (Fig. 4). Total 7,284 genes were annotated to GO terms compared with 6,566. The number of entries under molecular function increased from 14 to 15 while that for cellular component rose from 12 to 14. By contrast, the number of entries associated with biological process decreased from 17 to 16. In the cellular component category, the highest proportion of genes were assigned to the cell and membrane terms, followed by organelle, extracellular region part, and macromolecular complex. For molecular function, catalytic activity and binding were the most enriched terms. Other abundant functions included transporter activity, structural molecule activity, and signal transducer activity. Within the biological process category, metabolic process and cellular process dominated. Other notable terms included response to stimulus, developmental process, and multi-organism process.

**Fig. 5.**
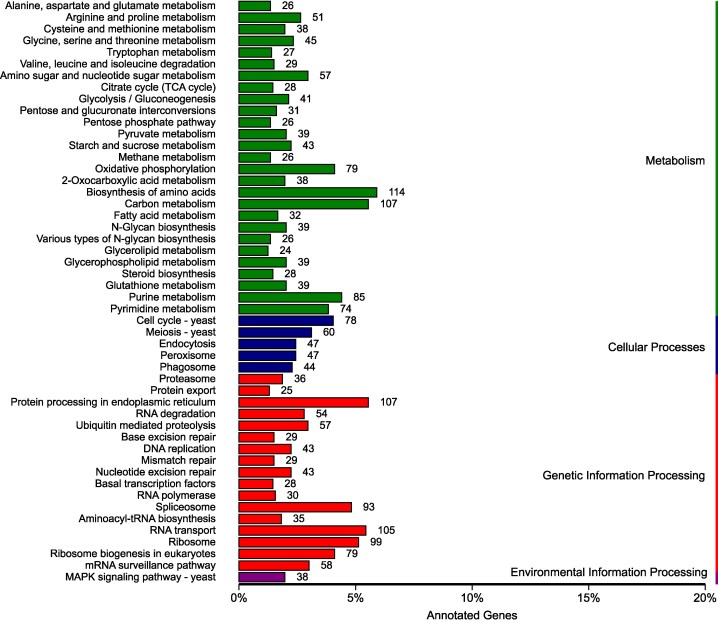
Classification statistics of KEGG pathway annotation. Distribution of genes in different KEGG categories.

KEGG pathway analysis annotated 3,365 genes from the newly assembled genome into 25 functional categories, representing an increase from the 3,254 genes annotated in the earlier genome. KEGG annotations were grouped into 4 major categories, genetic information processing, metabolism, cellular processes, and environmental information. Within metabolism, the most abundantly were biosynthesis of amino acids and carbon metabolism. Genetic Information Processing pathways were also highly enriched. The most prominent terms were protein processing in the endoplasmic reticulum, RNA transport, and ribosome. Within Cellular Processes, the most represented pathways were cell cycle—yeast and meiosis—yeast. Only 1 pathway, MAPK signaling pathway—yeast, was annotated under Environmental Information Processing (Fig. 5). Overall, the increased number of genes annotated to GO and KEGG reflects the improved quality of the genome assembly. The more comprehensive characterization of gene functions and metabolic pathways provides a solid foundation for further functional studies in *P. ostreatus* 00389.

### Prediction and distribution analysis of gene clusters

Gene cluster prediction was added to the new assembled genome, owing to improved sequencing technology and the enhanced integrity and continuity of the genome (Yu et al. 2023). A total of 35 gene clusters, spanning 1.09 Mb, was identified across 11 principal genomic scaffolds. Gene clusters in the new genome exhibit significant differences in number, length, and chromosome distribution. For example, the length of the gene clusters range from 14.10 (r6c3) to 64.96 kb (r9c1). Thirteen clusters were longer than 40 kb (e.g. r2c5, r9c1, r11c1), and 9 clusters were shorter than 20 kb (e.g. r6c3, r6c1, r1c2) (Table 10). This feature is consistent with the diversity pattern of fungal metabolic gene clusters reported in previously studies (Rokas et al. 2018). In addition, the distribution of these gene clusters varied substantially across different groups. Lachesis_group5 harbored 6 gene clusters, while Lachesis_group3 and Lachesis_group7 each contained only 1 cluster. A similar cluster distribution pattern was reported in edible and medicinal fungi such as *L. sulphureus* (Dong et al. 2022). Nevertheless, the functions of these gene clusters require additional experimental validation.

**Table 10.** Distribution and quantity of gene clusters on different groups.

Gene_cluster	scaffold_id	Start	End	Length (bp)
r1c1	Lachesis_group0	2,983,045	3,000,826	17,782
r1c2	Lachesis_group0	3,096,387	3,113,243	16,857
r1c3	Lachesis_group0	4,070,727	4,093,552	22,826
r2c1	Lachesis_group1	770,181	809,441	39,261
r2c2	Lachesis_group1	1,644,883	1,686,477	41,595
r2c3	Lachesis_group1	1,883,223	1,922,137	38,915
r2c4	Lachesis_group1	3,513,590	3,534,714	21,125
r2c5	Lachesis_group1	4,270,007	4,314,307	44,301
r3c1	Lachesis_group2	664,899	684,203	19,305
r3c2	Lachesis_group2	1,125,591	1,148,622	23,032
r3c3	Lachesis_group2	1,221,034	1,240,966	19,933
r3c4	Lachesis_group2	2,574,813	2,618,432	43,620
r3c5	Lachesis_group2	2,673,810	2,709,994	36,185
r4c1	Lachesis_group3	363,713	407,904	44,192
r5c1	Lachesis_group4	605,058	625,387	20,330
r5c2	Lachesis_group4	633,776	654,851	21,076
r6c1	Lachesis_group5	1,399,459	1,415,236	15,778
r6c2	Lachesis_group5	1,458,352	1,475,466	17,115
r6c3	Lachesis_group5	1,802,213	1,816,309	14,097
r6c4	Lachesis_group5	1,837,377	1,854,881	17,505
r6c5	Lachesis_group5	2,738,879	2,785,079	46,201
r6c6	Lachesis_group5	2,789,988	2,811,156	21,169
r7c1	Lachesis_group6	728,513	749,707	21,195
r7c2	Lachesis_group6	2,019,143	2,062,124	42,982
r8c1	Lachesis_group7	267,642	289,422	21,781
r9c1	Lachesis_group8	1,374,829	1,439,787	64,959
r9c2	Lachesis_group8	1,447,307	1,490,781	43,475
r9c3	Lachesis_group8	1,704,244	1,752,160	47,917
r9c4	Lachesis_group8	1,756,067	1,800,636	44,570
r10c1	Lachesis_group9	1,044,890	1,089,161	44,272
r10c2	Lachesis_group9	1,253,138	1,270,377	17,240
r10c3	Lachesis_group9	1,919,179	1,940,330	21,152
r11c1	Lachesis_group10	1,386,284	1,437,095	50,812
r11c2	Lachesis_group10	1,724,634	1,768,811	44,178
r11c3	Lachesis_group10	2,033,773	2,055,177	21,405

### Protein subcellular localization and annotation

Subcellular localization analysis of proteins was also performed for the new genome. As an important part of functional annotation, it serves as a core strategy for functional genomics research (Donnes and Hoglund 2004). A total of 1,336 signal peptide-containing proteins, 2,393 transmembrane proteins, 900 secreted proteins and 123 effector proteins were identified (Table 11). These secreted proteins encode diverse enzymes, including various GHs, polysaccharide lyases, carbohydrate esterases, and AAs (as shown in the CAZy annotation results). These enzymes were essential for lignocellulose decomposition (Floudas et al. 2012), indicating that this species possesses a complete enzyme system for lignocellulose degradation. The protein subcellular localization provides valuable insights into the biological functions of corresponding proteins (Fang et al. 2009). The identification of these functional proteins also lays a solid foundation for further exploring their molecular mechanisms in *P*. *ostreatus* 00389

**Table 11.** Protein subcellular localization results.

Protein type	Number
Signal peptide	1,336
Transmembrane protein	2,393
Secreted protein	900
Effector protein	123

### Conclusions

The genome of *P. ostreatus* strain CCMSSC 00389 was sequenced using the PacBio Sequel II platform, and Hi-C technology was applied to assist with chromosome-level genome assembly. Compared with the previous version, the newly assembled genome exhibited improvements in sequencing quality, contiguity and completeness. The total genome size increased from 35.82 to 40.39 Mb. In contrast, the number of scaffolds decreased from 2,529 to 20, with scaffold N50 length increased from 394.8 kb to 3.76 Mb. The newly assembled genome showed greatly prolonged read length and reduced data redundancy. Approximately 99.62% of the assembly were anchored onto 12 pseudo-chromosomes. Collinearity analysis revealed high synteny between the new and previous genomes versions. The completeness of the assembly was estimated to be 92.76% based on BUSCO in the new assembly, confirming the acceptable completeness of the new assembled genome. The proportion of the repetitive sequences increased from 0.04 to 14.02%, with improved annotation integrity for repetitive regions and transposable elements. The total number of protein-coding genes decreased from 13,438 to 12,964, whereas the total gene length, average gene size and exon number increased. Concurrently, the numbers of both noncoding RNAs and pseudogenes increased. These changes collectively reflected the optimization of genome composition and structure. The number of functionally annotated protein-coding genes increased from 11,095 to 12,514. This chromosome-level genome with refined structural integrity and comprehensive functional annotation provides a valuable genomic resource for future studies on the genetic mechanisms, functional gene validation, and molecular breeding of *P. ostreatus*.

## Supplementary Material

jkag150_Supplementary_Data

## Data Availability

The previous Illumina-based genome assembly of 00389 has been deposited in NCBI (Sayers et al. 2025) under accession number MAYC00000000.1 (https://www.ncbi.nlm.nih.gov/datasets/genome/GCA_001956935.1/), with the BioProject ID PRJNA327267 and BioSample ID SAMN05327826. The newly assembled genome of 00389 based on PacBio sequencing data reported in this paper has been deposited in the Genome Warehouse at the National Genomics Data Center (NGDC; Ma et al. 2025), China National Center for Bioinformation/Beijing Institute of Genomics, Chinese Academy of Sciences, under accession number GWHJIFT00000000.1 (https://ngdc.cncb.ac.cn/gwh/Assembly/129531/show), with the BioProject ID PRJCA062908 and BioSample ID SAMC7883800. The raw PacBio and Hic data of 00389 have been deposited in the Genome Sequence Archive of NGDC with the accession number CRA044537. Supplemental material available at G3 online.
