# Supplementary material for: Improved chromosome-level genome assembly of Pleurotus ostreatus CCMSSC 00389
Source: G3 (Bethesda). 2026 Jun 11;16(8):jkag150. doi: 10.1093/g3journal/jkag150 (PMC13439954; doi:10.1093/g3journal/jkag150)
Supplement: Supplementary file 1 [file jkag150_supplementary_data.zip › Figure_S1_G3-2026-406806.docx]

**Figure S1. Heat map of chromosome interaction in Hi-C assembly.** LG00~LG11 stands for Lachesis Group00~11. The abscissa and ordinate each represent the order of each bin on the corresponding chromosome group.
